# Neural Plasticity in Functional and Anatomical MRI Studies of Children with Tourette Syndrome

**DOI:** 10.3233/BEN-120294

**Published:** 2013

**Authors:** Heike Eichele, Kerstin J. Plessen

**Affiliations:** ^1^Department of Biological and Medical PsychologyFaculty of PsychologyUniversity of BergenBergenNorway; ^2^Department of Clinical MedicineFaculty of Medicine and DentistryUniversity of BergenBergenNorway; ^3^K.G. Jebsen Centre for Research on Neuropsychiatric DisordersUniversity of BergenBergenNorway; ^4^Center for Child and Adolescent Psychiatry BispebjergCapital Region PsychiatryBispebjergDenmark; ^5^Institute for NeurologyPsychiatryand Sensory SciencesUniversity of CopenhagenCopenhagenDenmark

**Keywords:** Tourette syndrome, children, Magnetic Resonance Imaging, neural plasticity, adaptation

## Abstract

**Background:** Tourette syndrome (TS) is a neuropsychiatric disorder with childhood onset characterized by chronic motor and vocal tics. The typical clinical course of an attenuation of symptoms during adolescence in parallel with the emerging self-regulatory control during development suggests that plastic processes may play an important role in the development of tic symptoms.

**Methods:** We conducted a systematic search to identify existing imaging studies (both anatomical and functional magnetic resonance imaging [fMRI]) in young persons under the age of 19 years with TS.

**Results:** The final search resulted in 13 original studies, which were reviewed with a focus on findings suggesting adaptive processes (using fMRI) and plasticity (using anatomical MRI). Differences in brain activation compared to healthy controls during tasks that require overriding of prepotent responses help to understand compensatory pathways in children with TS. Along with alterations in regions putatively representing the origin of tics, deviations in several other regions most likely represent an activity-dependent neural plasticity that help to modulate tic severity, such as the prefrontal cortex, but also in the corpus callosum and the limbic system.

**Discussion:** Factors that potentially influence the development of adaptive changes in the brain of children with TS are age, comorbidity with other developmental disorders, medication use, IQ along with study-design or MRI techniques for acquisition, and analysis of data. The most prominent limitation of all studies is their cross-sectional design. Longitudinal studies extending to younger age groups and to children at risk for developing TS hopefully will confirm findings of neural plasticity in future investigations.

## 1. Introduction

Tourette syndrome (TS) is a childhood onset neuropsychiatric disorder defined by the presence of multiple motor tics and at least one vocal tic for more than one year [1] and with a prominent genetic influence [44]. Motor tics usually manifest between the age of 3 and 8 years [31], most commonly as eye blinking, facial grimacing, or nose twitching. Vocal tics typically follow the onset of motor tics by several years [57] and often present themselves initially by coughing, throat clearing or the production of short meaningless sounds. Tic disorders show a typical course in most persons

ISSN 0953-4180/13/$27.50 © 2013 – IOS Press and the authors. All rights reserved affected with the disorder. Tic severity often increases until puberty and subsequently declines such that about 40% of children are tic-free at the age of 18 [33]. This decline in symptoms coincides with the development of self-regulatory control during childhood and adolescence [11,69], which is accompanied by the maturation of the frontal cortex [19].

Motor and vocal tics fluctuate in severity, intensity and frequency [56], and both external and physiological factors influence the expression of tics. Recent evidence thus conceptualizes tics as semi-voluntary habits, rather than involuntary movements [23]. Tics are often preceded by "premonitory urges", which are unpleasant bodily sensations, acting as internal cues for the execution of a tic [31]. These sensory phenomena consist of a physical experience of tension and prompt a moment of relief after performing the tic. The reduction of this unpleasant sensory experience thus may represent the underlying incentive for the completion of a tic, resulting in a negative reinforcement circuit that supposedly facilitates habit learning. FMRI studies suggest that those urges correlate with increased brain activity within the insula and the cingulate motor areas [5,27] and contribute to tic generation within the cortico-striatal-thalamo-cortical circuits (CSTC) [35,72].

Suppression of tics may be regarded as the repeated overriding of an automatic behavioral response to the premonitory urge. A promising behavioral treatment approach, Habit Reversal Training (HRT), makes use of the fact that in many instances premonitory urges cue the execution of tics [49]. HRT employs the ability of individuals with TS to actively suppress tics while performing a competing motor response. However, the improvement of tic-symptoms during puberty even without the above mentioned intervention suggests that individuals with TS relentlessly and in most instances unconsciously aim to suppress emerging tics to improve their psychosocial function. Multiple studies report superior cognitive control abilities in children with pure TS as compared to controls [25,26,42,62], suggesting a transfer of enhanced behavioral control to other cognitive domains.

Functional and anatomical imaging in TS serve as a window into the developing brain and contribute to the understanding of trajectories of brain development in children with neuropsychiatric disorders. More detailed knowledge concerning underlying neurobiological circuits involved in the disorder will contribute to the development of specific treatment approaches focusing on the facilitation of adaptive and plastic processes with the overall aim to reduce symptoms. Functional imaging of the brain at rest or during tasks relevant to tic suppression can reveal which parts of the brain are involved in the adaptive processes of individuals with TS. A few MR studies in humans have reported direct evidence of plastic changes in anatomic regions as a result of repeated training activity [6,14], setting the framework for mapping neural plasticity in children with TS by means of anatomical MRI. The majority of early imaging studies in TS, however, focused on adult participants, even though the typical course in TS suggests that only a minority of patients retain their tic symptoms through adulthood. Adults with TS thus may represent a selected sample, due to the absence of behavioral adaption in this group.

Herein, we chose to focus on existing functional and anatomical MRI studies, although other modalities, such as electroencephalography, magnetic encephalography and transcranial magnetic stimulation have in recent years likewise added interesting evidence of the plastic changes in individuals with TS. The existing studies using those modalities, however, have largely examined adults. We will provide a review of findings derived from functional and anatomical imaging studies in children with TS to identify brain areas and processes involved in the development of neural plasticity, along with important sampling characteristics of the studied populations, such as their age, prevalence of comorbid illnesses, and IQ – all factors that are possibly relevant to a more or less successful adaptation to tics.

## 2. Methods

We limited the scope of this review to functional and anatomical MRI studies and performed the search using Pubmed (ncbi.nlm.nih.gov/pubmed), ISI Web of Knowledge (apps.isiknowledge.com), and Embase (http://ovidsp.uk.ovid.com) databases. Combined search terms were (Magnetic Resonance Imaging, MR, MRI, Neuroimaging) AND (Tourette Syndrome, TS, tics, tic disorder) AND (neural plasticity, plasticity, compensatory, neuroplastic, adaptive). Performing this search in April 2012 resulted in 35 original studies that were published or published online ahead of print. The authors screened the abstracts of the initial search results for relevance, and used the reference lists of published articles to identify possible additional studies. The following inclusion criteria were applied: (a) results were published within the last 15 years, due to technical differences in the MRI studies (e.g. resolution of the MR-images, magnetic field strength) in children with TS before this date (b) language of the publication was English, French, Norwegian, Swedish, Danish or German, (c) included a group of at least 10 participants with the mean age being younger than 19 years. Findings from the 13 studies meeting inclusion criteria were categorized by the different approaches utilized: Structural MRI, functional MRI, and Diffusion Tensor Imaging (DTI) (Table 1).

## 3. Results

### *3.1. Functional MRI*

#### *3.1.1. Motor activity*

Several studies (Table 1) indicate that individuals with TS exhibit an abnormal organization of the motor system, especially in the basal ganglia (for reviews see [18,39,50]). Functional imaging studies of finger tapping tasks can help to identify pathology affecting the motor system. In a recent fMRI study [61], 19 medication-naïve boys with TS and without comorbid conditions showed less activation in the left precentral gyrus in TS compared with the healthy control children in a right index finger tapping paradigm. Boys with TS displayed higher activation in the left caudate nucleus and the right medial frontal gyrus. The authors concluded that alterations in those brain regions could represent signs of early compensation within the functional organization of the motor execution network.

#### *3.1.2. Interference tasks*

Interference tasks have been used in several fMRI studies in children with TS because these tasks are similar to the everyday experience of modulating an automatic response (i.e., the tic) to allow for another more appropriate behavior (i.e., the task they are performing). An interference task introduces a response conflict between a correct response based on an abstract, pre-learned mapping rule and an incorrect, but prepotent response triggered, for example, by spatial compatibility between the position of the responding hand and the stimulus as in the Flanker and Simon tasks [55]. Functional imaging allows for the identification of task-related changes in activity and connectivity in brain networks involved in self-regulatory control and the mechanisms that presumably lead to the structural reorganization in the brain of children with TS.

In a Stroop interference task including 32 children with TS and 20 healthy control children (and 34 adults with TS and 50 healthy control adults), participants with TS and healthy controls did not differ in their behavioral performance, but did show different spatial patterns of brain activation [37]. Activations in ventral prefrontal and posterior cingulate cortices showed a prominent interaction between group and age. Post-hoc analyses revealed that individuals with TS, in contrast to controls, showed a tendency to deactivate those regions less and these deactivations further decreased with age. These regions coincided with the default-mode network (DMN). In addition, participants with more severe tics showed increased activation within the dorsolateral prefrontal cortex. The authors concluded that participants with TS overactivated these regions to maintain task performance.

In another fMRI study including 18 unmedicated children with TS compared to 19 controls, tic severity was correlated with slower performance in the most demanding task condition of a cognitive control task involving rule manipulation, task-switching manipulation and interference suppression, indicating that the task was more difficult for individuals with more severe tics [3]. FMRI analyses revealed that greater activation of the dopaminergic substantia nigra/ventral tegmental area and cortical, striatal, subthalamic and thalamic regions in the CSTC circuits correlated with higher tic severity. Finally, participants with TS engaged their prefrontal cortices more while performing the task, compared with the control group, possibly indicating compensatory activation.

A third fMRI study, using the the spatial interference-based Simon task, testing spatial interference, compared brain activation in 22 children with TS with healthy controls in the same age range and with adults in the two groups [54]. Task-relevant activations were more widespread in the younger participants, whereas older individuals showed more specific fronto-striatal activation, indicating a general age-related shift. These age-related differences were more pronounced in the TS group despite comparable performance in the task. In addition, tic severity correlated positively with frontal activation, supporting the authors' suggestion that activation of fronto-striatal circuits supports both efficient performance and regulation of tic severity. Further, the authors suggested that adults with persistent TS may have deficiencies in these circuits due to a failure of prefrontal plasticity and disturbances in striatal function.

Finally, in a manual task-switching paradigm, 10 children with TS showed greater activation in the right prefrontal cortex at the same level of performance as compared to 15 control children [26]. In addition, the greater activation was strongly related to comparable behavioral performance in children with TS and controls, indicating enhanced cognitive control of motor output during a behavioral task with a high level of intermanual conflict.

*3.1.3. Inhibitory tasks*

The ability to suppress a prepotent response is an important function for executing control. Response inhibition allows appropriate responses to meet complicated task demands and adaptation to changing environments [71]. The most common measures of response inhibition are the go/no-go and stop-signal response time task. In these behavioral tasks, the dominant or more frequent stimulus constitutes a 'go' signal requiring the subjects to respond within a time window and therefore establishes a prepotent response tendency. While there have been no imaging assessments of inhibitory function in childhood TS are available on these canonical response inhibition tasks, a recent fMRI study has examined blink suppression in TS, since premonitory urges in TS are often compared to the feeling of not being allowed to blink [38]. In this study, 22 children with TS were compared with 16 healthy children (along with 16 adults with TS and 17 healthy control adults) while inhibiting their urge to blink. Children with TS showed higher activation in the frontal cortex and striatum during eye blink inhibition. Activation increased more with age in regions of the dorsolateral and inferolateral prefrontal cortex and caudate nucleus in participants with TS. Furthermore, participants with TS showed more activation in the middle frontal gyrus, dorsal anterior cingulate, and temporal cortices. Tic severity correlated inversely with activation in putamen and inferolateral prefrontal cortex.

*3.1.4. Resting state activity*

Resting state networks describe spatially organized and temporally correlated brain activity when participants are at rest and not performing a specific task. Resting state networks include the default mode network, which is often anticorrelated to task-related networks and may therefore represent the systems readiness to engage in interference task [17]. Those networks mature during childhood [16] and may express a factor for the level of maturation of the central nervous system [13]. Resting-state fMRI in 33 adolescents with TS (aged 9–15) revealed immature patterns of functional connectivity, especially in two networks involved in task cognitive/executive control [8]. The fronto-parietal network is more likely involved in more rapid, adaptive online control, and a cingulo-opercular network that is important for self-maintenance. The developmental transitions seen during maturation in healthy children did not appear in TS, and the brains of children with TS appeared younger than their chronological age. Most differences between groups were present in fronto-parietal networks; individuals with TS showed weaker connectivity between distant areas of the brain, but stronger connectivity between neighboring areas of the brain. On the basis of these results, the authors argued that differences in patterns of connectivity are adaptive effects to support online task-control and to compensate for the inability to suppress tics.

*3.2. Anatomical MRI and Diffusion Tensor Imaging*

Evidence derived from clinical [39], neuropsychological [36] and imaging studies point to the basal ganglia as the region in the brain exhibiting the genetic vulnerability for TS. Several anatomical MRI investigations, and in particular one large study of basal ganglia volumes in children and adults with TS and healthy control subjects [48], revealed smaller volumes of the nucleus caudate bilaterally in children and adults with TS as compared to healthy controls. The presence of significantly smaller caudate nuclei in both age groups suggests that reduced volumes of the caudate nucleus may represent a trait morphological deviation in persons with TS. Moreover, the persistence of decreased volumes of the caudate nucleus into adulthood implies that the caudate nucleus is not a prime target for adaptive changes in response to the presence of tics. The predictive role of the volume of the caudate nucleus was confirmed in a follow-up study of a large sample of children with TS, revealing that the size of the caudate nucleus in childhood correlated inversely with the severity of tics and with Obsessive Compulsive Disorder (OCD) symptoms in early adulthood [4].

Even though these studies shed light on the origin of tic behavior, other circuits are involved in the regulation of tic behavior. It is the interaction of striatal, limbic and cortical portions of the CSTC circuits that largely determines an individual's capacity for self-regulatory control, such as the ability to inhibit unwanted impulses, to monitor ongoing actions or to plan future actions [10]. These circuits are thus of high relevance for successful modulation of tic-behaviors. In line with this notion, a study using high-resolution MRI (Table 2) showed that regional volumes of the

Table 1

Functional MRI studies indicating plasticity in children with TS

| | Area of interest | Tourette (N); age (SD); age range; gender (male/female) | Controls (N); age (SD) | Comorbidity | Tic severity | IQ (Test), mean (SD) | Medication | Method | Main findings |
|---|---|---|---|---|---|---|---|---|---|
| Motor activity | | | | | | | | | |
| Roessner, Wittfoth et al. 2012 [61] | Whole brain /ROI | $N = 19$; 12.5 years (1.4); age range n.a.; 19/0 | $N = 16$; 12.9 years (1.6) | No comorbidity | TSSS score = 1.9, SD = 1.4 | IQ (subtests of WISC-Revised), full-scale IQ TS = 106.1 (13.7), controls = 106.3 (10.7) | None | fMRI/finger tapping | TS: ↓ activation in left precentral gyrus, ↑ activation in caudate nucleus and medial prefrontal cortex |
| Interference task | | | | | | | | | |
| Marsh, Zhu et al. 2007 [37] | ROI | Children $N = 32$, adults $N = 34$; children 12.8 years (2.8), adults 35.27 years (11.1); children 8.4–17.5 years, adults 17–52.6 years; children 27/5, adults 20/14 | Children $N = 20$ adults $N = 50$; children 13.5 years (3.2), adults 31.9 years (11.4) | OCD $N = 10$, ADHD $N = 6$, OCD+ADHD $N = 4$ | YGTSS motor tic present = 18.5, YGTSS lifetime-w.e. children = 28.41, YGTSS phonic tic score present children = 10.2, YGTSS phonic w.e. children = 9.9 | IQ (WASI), full-scale IQ TS = 113.9 (13.2), controls = 117.5 (12.3) | Stimulants $N = 3$, Haloperidol/pimozide $N = 6$, Risperidone $N = 5$, $\alpha$-adrenergic agonists $N = 6$, SSRI $N = 8$, | fMRI/Stroop interference task | TS: failed to ↓ activity in mesial prefrontal cortex, in ventral anterior cingulate cortex, in posterior cingulate cortex with age, failed to ↑ activity in fronto-striatal circuits with age |
| Baym, Corbett et al. 2008[3] | Whole brain /ROI | $N = 18$; 10.42 years; 7–13 years; 15/3 | $N = 19$; 10.33 years | OCD $N = 8$, ADHD $N = 59$ | YGTSS mean score = 21, SD = 7 | Exclusion IQ (WASI) below 75, estimated IQ TS = 106 (14), controls = 117 (12) | Atomoxetine $N = 2$, off medication > 40 hours before scanning | fMRI/ cognitive control task | ↑ Tic severity correlated to ↓ performance, ↑ tic severity correlated to ↑ activation of dopaminergic nuclei and cortical, striatal and thalamic regions; ↑ tic severity associated with ↑ engagement of subthalamic nucleus |
| Raz, Zhu et al. 2009 [54] | Whole brain | Children $N = 22$, adults $N = 16$; children 13.0 years (2.7), adults 38.1 years (13.5); children 8.9–18.2, adults 23.9–64.6; children 12/10, adults 10/6 | Children $N = 16$, adults $N = 17$; children 12.8 years (2.6), adults 40.4 years (12.4) | OCD+ADHD $N = 1$ | YGTSS mean score = 13.0, SD = 2.7 | Exclusion IQ (WASI) below 75, full-scale IQ TS = 113.9 (13.2), controls = 117.5 (12.3) | n.a. | fMRI/Simon task | TS: pronounced ↑ specific frontostriatal activation with age, correlation tic severity with ↑ frontal activation |
| Jackson, Parkinson et al. 2011 [26]* | ROI | $N = 10$, 13.75 years (1.75 years); 10.8–18.08 years ($N = 13$); 8/2 | $N = 15$; 14 years, 4 months (1 year 11 months) | No comorbidity | YGTSS global Yale score present = 31.7, SD = 21.3 for $N = 13$ | IQ (WASI), estimated IQ TS = 106.6 (SD) = 14.7 for $N = 13$, controls n.a. | Clonidine $N = 5$, Risperidone $N = 1$, Fluoxetine $N = 1$, Melatonin $N = 1$, Aripiprazole $N = 1$ for $N = 13$, 1 TS participant > 1 med. | DTI and fMRI (task-switching paradigm) | TS: ↑ BOLD response in right prefrontal cortex during high-conflict trials |
| Inhibitory task | | | | | | | | | |
| Mazzone, Yu et al. 2010 [38] | Whole brain /ROI | Children $N = 22$, adults $N = 29$; children 13.1 years (2.6), adults 35.1 years (11.1); age range n.a.; children 19/3, adults 17/12 | Children $N = 21$, adults $N = 48$; children 13.4 years (3.1), adults 31.4 years (11.0) | OCD $N = 21$, ADHD $N = 11$, OCD+ADHD $N = 4$ | YGTSS mean score present = 18.5, YGTSS lifetime-w.e. score children = 28.9 | Exclusion IQ (WASI) below 75, full-scale IQ TS children = 116.0 (12.1), controls children = 116.1 (13.4) | Typical neuroleptics $N = 5$, risperidone $N = 4$, $\alpha$-adrenergic agonists $N = 2$, SSRI $N = 11$, levothyroxine $N = 2$, valproic acid $N = 2$ | fMRI/eye blinking inhibition | TS: fronto-striatal activation ↑ regions during inhibition |

Table 1, continued

| | Area of interest | Tourette (N); age (SD); age range; gender (male/female) | Controls (N); age (SD) | Comorbidity | Tic severity | IQ (Test), mean (SD) | Medication | Method | Main findings |
|---|---|---|---|---|---|---|---|---|---|
| Resting state | | | | | | | | | |
| Church, Fair et al. 2009 [8] | Functional connectivity in 39 previously defined putative regions during resting state | $N = 33$; 12.7 years (SD n.a.); 9.92–15.83 years; 25/8 | $N = 42$; 12.69 years (SD n.a.) | OCD $N = 21$, ADHD $N = 11$, OCD+ADHD $N = 4$ | YGTSS mean score = 16.3 | Exclusion IQ (WISC-IV) below 80, full-scale IQ TS children = 105.4 (SD n.a.), controls children = 108.5 (SD n.a.) | Alpha2-agonists $N = 13$, atypical neuroleptics $N = 7$, stimulants $N = 7$, SSRI $N = 6$, benzodiazepines $N = 2$, antiepileptic meds. $N = 2$, norepinephrine RI antidepressants $N = 2$, beta-blockers $N = 1$, tetracyclic antidepressants $N = 1$, 13 TS participants > 1 med | fMRI/resting state, functional connectivity | TS: failure to develop age-appropriate networks |

"↑" = Increased; "↓" = Decreased; ADHD = Attention-deficit/hyperactivity disorder; BG = Basal ganglia; DLPF = Dorsolateral prefrontal; DTI = Diffusion Tensor Imaging; ROI = Region of interest measurement; SSRI = selective serotonin reuptake inhibitor; TS = Tourette syndrome; TSSS = Tourette Syndrome Severity Scale; VBM = Voxel-based morphometry; VOI = Volume of interest measurement; WASI = Wechsler Abbreviated Scale of Intelligence; WISC = Wechsler Intelligence Scale for Children; WBV = Whole brain volume; YGTSS = Yale Global Tic Severity Scale; YGTSS-we = Yale Global Tic Severity Scale-worst ever score. *Jackson, Parkinson et al. [26] includes a functional and an anatomical study and appears therefore in Table (1) and (2).

Table 2

Anatomical MRI studies indicating plasticity in children with TS

| | Area of interest | Tourette (N); age (SD); age range; gender (male/female) | Controls (N); age (SD) | Comorbidity | Tic severity | IQ (Test), mean (SD) | Medication | Method | Main findings |
|---|---|---|---|---|---|---|---|---|---|
| Cortical regions | | | | | | | | | |
| Peterson, Staib et al. 2001 [47] | Cortical regions, ventricles | $N = 155$; 18.7 years (13.4); 6–63 years; 114/41 | $N = 131$; 20.8 years (13.4) | OCD $N = 62$, ADHD $N = 36$ | YGTSS scores n.a | Exclusion IQ below 80, IQ scores n.a. | Stimulants $N = 3$, traditional neuroleptics $N = 20$, risperidone $N = 7$, $\alpha$-adrenergic agonists $N = 29$, SSRI $N = 19$, tricyclics $N = 11$ | Parcellation of cerebral subdivisions | TS: dorsal prefrontal volumes and parieto-occipital regions ↑, inferior occipital volumes ↓ |
| Corpus callosum | | | | | | | | | |
| Plessen, Wentzel-Larsen et al. 2004 [53] | Corpus callosum | $N = 158$; 18.5 years (13.3); 5–65 years; 117/41 | $N = 121$; 19.7 years (12.6) | OCD $N = 39$, ADHD $N = 32$, OCD+ADHD $N = 10$ | YGTSS motor tic present = 11.4, SD = 4.6, Adults mean = 14.9, SD = 4.2, YGTSS lifetime-we-children = 15.0 SD = 4.3, versus adults = 18.8, SD = 2.7, YGTSS phonic we children = 11.6, SD = 4.5, versus adults 14.4, SD = 4.4; | Exclusion IQ below 80, IQ scores n.a. | Traditional neuroleptics $N = 12$, risperidone $N = 4$, $\alpha$-adrenergic agonists $N = 22$, SSRI $N = 12$, tricyclics $N = 9$, combination of traditional neuroleptics and SSRI $N = 4$, other $N = 1$ | ROI | Interaction with age: TS children having ↓ CCs and TS adults ↑ CCs |
| Plessen, Gruner et al. 2006 [51] | Corpus callosum | $N = 20$; 13.6 years (1.9); 9–17 years; 20/0 | $N = 20$; 13.4 years (2.4) | OCD $N = 4$, ADHD $N = 5$, OCD+ADHD $N = 0$ | YGTSS motor tic present = 11.4, SD = 2.9, YGTSS lifetime-we = 15.5 SD = 5.1, YGTSS phonic tic score present = 9.2, SD = 3.4, YGTSS phonic we = 13.7, SD = 5.5 | Exclusion IQ (WISC-III) below 70, full-scale IQ TS = 94.5 (10.2), controls = 105.7 (9.2) | Neuroleptics $N = 4$, $\alpha$-adrenergic agonists $N = 2$, SSRI $N = 1$, stimulants $N = 2$, | DTI analysis of CC subdivisions | TS: ↓ FA values in all subdivisions of the CC |
| Roessner, Overlack et al. 2011 [60] | BG, thalamus, corpus callosum and its subregions | $N = 49$; 12.0 years (1.3); 9–15 years; 49/0 | $N = 42$; 12.1 years (1.3) | No comorbidity | TSSS scores = 2.2, SD = 1.5 | IQ (WISC-R), full-scale IQ TS = 109 (13), controls = 110 (11) | None | Volumetric analysis | TS: ↑ left and right putamen, ↑ subregion3 of the CC |
| Jackson, Parkinson et al. 2011 [26]* | Whole brain with focus on white matter | $N = 14$; 14.5 years (SD 2.5 years) for $N = 13$; 10.8–18.08 years for $N = 13$; 12/2 | $N = 14$; 15.3 years (SD n.a.) | No comorbidity | YGTSS present = 31.7, SD = 21.3 for $N = 13$ | IQ (WASI), estimated IQ TS = 106.6 (SD) = 14.7 for $N = 13$, controls n.a. | Clonidine $N = 5$, Risperidone $N = 1$, Fluoxetine $N = 1$, Melatonin $N = 1$, Aripiprazole $N = 1$ for $N = 13$, 1 TS participant > 1 med. | DTI and fMRI | TS: differences in microstructure, including CC and forceps minor, correlation with tic severity |
| Thalamus | | | | | | | | | |
| Miller, Bansal et al. 2010 [40] | Thalamus and subregions | $N = 149$; 18.7 years (13.0); 6–63 years; 112/37 | $N = 134$; 22.9 years (13.7) | ADHD $N = 42$, OCD $N = 55$, OCD+ADHD $N = 10$ | YGTSS scores n.a. | Exclusion IQ (WASI) below 70, full-scale IQ TS = 113.8 (16.8), controls = 120.0 (17.1) | Typical neuroleptics $N = 15$, atypical neuroleptics $N = 7$, $\alpha$-adrenergic agonists $N = 28$, SSRI $N = 20$, stimulants $N = 5$, tricyclics $N = 12$ | VOI and surface matching analysis | TS: overall volumes thalamus ↑, locally ↑ over lateral thalamus |
| Amygdala&Hippocampus | | | | | | | | | |
| Peterson, Choi et al. 2007 [45] | Amygdala and hippocampus | $N = 154$; 18.7 years (13.4); 6–63 years; 115/39 | $N = 128$; 20.2 years (13.2) | OCD $N = 48$, ADHD $N = 42$, OCD+ADHD $N = 10$ | YGTSS scores n.a. | Exclusion IQ (Kauffmann Brief Intelligence Test) below 70, full-scale IQ TS = 113 (16.1), Controls = 119.5 (16.9) | Typical neuroleptics $N = 19$, atypical neuroleptics $N = 7$, $\alpha$-adrenergic agonists $N = 28$, SSRI $N = 20$, stimulants $N = 3$, tricyclics $N = 12$, YGTSS scores n.a. | VOI and surface matching analysis | TS: overall volumes amygdala and hippocampus ↑ |

"↑" = Increased; "↓" = Decreased; ADHD = Attention-deficit/hyperactivity disorder; BG = Basal ganglia; DLPF = Dorsolateral prefrontal; DTI = Diffusion Tensor Imaging; ROI = Region of interest measurement; SSRI = selective serotonin reuptake inhibitor; TS = Tourette syndrome; TSSS = Tourette Syndrome Severity Scale; VBM = Voxel-based morphometry; VOI = Volume of interest measurement; WASI = Wechsler Abbreviated Scale of Intelligence; WISC = Wechsler Intelligence Scale for Children; WBV = Whole brain volume; YGTSS = Yale Global Tic Severity Scale; YGTSS-we = Yale Global Tic Severity Scale-worst ever score. *Jackson, Parkinson et al. [26] includes a functional and an anatomical study and appears therefore in Table (1) and (2).

dorsal prefrontal and parietal cortex were significantly larger and inferior occipital volumes were significantly smaller in 155 children and adults with TS compared to 131 controls (age 6–63 years, 61.9% children). The association of larger cortical volumes in orbitofrontal and parieto-occipital regions with fewer tic symptoms confirmed the clinical significance of these findings, implying that the anatomical deviations represent a plastic reorganization of the prefrontal cortex to facilitate tic suppression [47]. The findings in children, however, differed markedly from those in adults with TS indicated by a strong interaction of Diagnosis x Age x Region, showing that dorsal prefrontal volumes were smaller in adults with TS compared to controls. Activity-dependent plasticity and hypertrophy within prefrontal regions could help to attenuate the severity of tic symptoms due to its capacity to increase the inhibitory reserve in the prefrontal regions and the capacity for self-regulatory control that is noted in the typical development of children and adolescents [11].

The corpus callosum (CC), the largest interhemispheric commissure, connects cortical regions in the brain and thus modulates and influences activity throughout the cortex. A study including 158 individuals ($n = 113 < 19$ years) with TS compared to 121 controls ($n = 66 < 19$ years) revealed that the size of the CC was smaller in children with TS and larger in adults with TS compared with healthy age-matched controls [53]. Furthermore, the size of the midsagittal CC correlated inversely with volumes of the prefrontal cortex in both the TS and control group, but the magnitudes of these inverse correlations was significantly higher in the TS group. Finally, the size of the CC correlated with the severity of motor tics, indicating that a smaller CC may have a protective or compensatory function in subjects with TS. In a region-of-interest Diffusion Tensor Imaging (DTI) study [51], the fractional anisotropy (FA) in the CC was significantly reduced throughout all partitions in 20 boys with TS (9–18 years) compared to 20 control children. FA is a measure of the integrity or the density of white matter, suggesting that its reduction speaks for an impaired interhemispheric connectivity. The authors have argued that a smaller CC, with a reduced interhemispheric connectivity may facilitate prefrontal function and thereby enhance the suppression of tics. This finding was corroborated in a recent study [26] conducting a whole-brain analysis of tract-based spatial statistics in 14 children with TS and 14 controls. Individuals with TS showed several tracts with reduced FA and increased mean diffusivity (MD), especially in the CC and the forceps minor. FA correlated with tic-severity, in line with the suggestion that the reorganization of the white matter in the CC may be a plastic response to improve prefrontal suppression of tics. Another recent study reported potentially contradictory results: 49 boys with TS showed a larger CC when compared to 42 controls (9–15 years), especially in the motor portion [60], which was considered a result of continuous motor activity due to the execution of tics, however, CC size did not correlate with tic-severity in that study.

The last modulatory station in the cortico-subcortical feedback circuit for self-regulatory control is the thalamus. High-resolution structural MR was used to examine both overall thalamic volume and local volumetric surface differences in a large sample of 149 individuals with TS ($n = 105 < 18$ years) and controls ($n = 58$ $< 18$ years) [40]. Such fine-grained analyses may help to localize volumetric differences to specific clusters of voxels on a structure's surface with more homogenous function [2]. Thalamic volume in children and adults with TS was overall enlarged and surface analysis revealed higher local volumes over the lateral thalamus, which is regarded as the anatomical cross-roads of activity-dependent hypertrophy or adaptive response within a larger compensatory system [21]. However, an interaction of Diagnosis x Sex indicated that female participants with TS showed a proportionally larger outward deformation. Finally, the inverse correlation of outward subregion with symptom severity was valid only for the female participants with TS.

Limbic portions of the cortical-subcortical circuits have been implicated in TS in one major study [45]. Using anatomical MRI, the authors reported that children with TS have an overall enlargement of hippocampus and amygdala in a large sample including 154 TS ($n = 109 < 18$ years) and 128 control subjects ($n = 72 <$ 18 years). Moreover, detailed morphometric analyses of the surface of both regions localized the volumetric differences mainly to the head and the distal tail bilaterally and left medial border of the hippocampus and the dorsal and ventral surfaces of the amygdala. The local volumes of these subregions on the surface of the structures declined with age in the TS group resulting in larger subregions in children, but smaller subregions in adults with TS, compared with the controls, where these strong correlations with age were absent. Finally, inverse correlations of those local volumes with tic severity suggested the adaptive and compensatory nature of the hypertrophy, in line with the hippocampal involvement in the extended networks of neural compensation and finally the intimate interaction of the hippocampus and the amygdala with prefrontal regions [7].

## 4. Discussion

We reviewed original studies using functional and anatomical MRI to improve the understanding of neuroplastic changes the brain of children with TS. This resulted in the identification of several complementary processes of neural plasticity involved in the execution and the modulation of tics.

First, most fMRI studies indicate the presence of adaptive compensatory processes at a functional level that help to maintain performance in the presence of an impaired motor function [3,37,54,61]. Evidence for underlying disturbances in motor circuits is consistent with reduced inhibitory interneurons in the striatum [28] and with the notion that individuals with TS show reduced inhibition of their motor cortices [73]. Moreover, motor activity is postulated as excessive within the basal ganglia's direct output pathway, which would in turn disinhibit thalamocortical projections and produce excess synaptic activity within the motor portion of the CSTC loops [41]. These compensatory processes involve the activation of the prefrontal, cingulate, striatal, and thalamic regions and are consistent with the role that these regions play in guiding the precision of motor movements, but also in conflict-evoking interference tasks.

Second, activity-dependent hypertrophy in motor areas has been shown to result in a plastic reorganization of parts of the brain that are directly involved in the performance of tics, such as excessive tic-related activity in motor circuits [60].

Third, compensatory reorganization appears to develop in brain regions involved in facilitating the successful modulation and suppression of tics. This continuous activity presumably results in an enlargement of the prefrontal cortex [47], the amygdala, and hippocampus [45], but a smaller CC [53], all of which were associated with less severe symptoms. These findings suggest a compensatory response that helps to attenuate symptoms by increasing the inhibitory reserve, consistent with the role of the dorsal prefrontal region in subserving self-regulatory functions of fronto-striatal circuits [67]. The importance of the prefrontal cortex for the suppression of tics has been documented in the first fMRI study in adults with TS, revealing significant activations in prefrontal and fronto-temporal regions and the caudate nucleus during periods of voluntary tic suppression compared with a rest condition, where participants were not suppressing their tics [46].

Finally, several studies imply that children with TS are superior to controls in cognitive control tasks, with enhanced self-regulatory control in a compensatory manner, which is, however, difficult to disentangle from the transfer seen in the brain e.g. as the result of cognitive training. Although the anatomical MRI studies in children with TS discussed here have not measured the capacity of self-regulatory control, several of the fMRI studies have used paradigms that involve cognitive control, where children with TS showed comparable [3,37] or better performance than controls [26]. The capacity for cognitive control in patients with TS is a topic of discussion and existing reports suggest conflicting results [9,15,43,59,65]. Several behavioral investigations also suggest that children with TS even may exert enhanced inhibitory control in situations that require cognitive control. Children with TS out-perform controls in directing their eye-movements [25,42]. In addition, because tic suppression activates the same prefrontal brain regions as tasks requiring cognitive control, parallel processes may be involved in performance monitoring and in tic-suppression [46].

### *4.1. Clinical characterization*

Several intra-individual variables may determine the capacity of a child to unconsciously learn to modulate tics, which in the long run, may lead to a plastic reorganization of the brain. The present studies, which have been selected for their focus on adaptive control and plasticity, may illuminate the significance of a few of these factors.

#### *4.1.1. Tic severity*

The evidence pointing toward underlying plastic processes is mostly based on correlations with tic-severity in the presented studies. The execution of many tics per day may trigger compensatory phenomena, but on the other hand, it may also represent the inherent lack of capacity to modulate the symptoms. Furthermore, the fact that tics wax and wane in their frequency and characteristics over hours, days, and months, makes the objective measurement of symptoms difficult. The studies selected in this review almost exclusively reported tic severity by using the Yale Global Tic Severity Scale (YGTSS) [32], with the exception of [61] who used the Tourette Syndrome Severity Scale (TSSS) [63]. Although the reported measurements differ with respect to reporting motor or vocal scores, either as "present" or "worst-ever" scores (see Tables 1 and 2), all selected studies demonstrated inverse correlations of anatomic or functional deviations with tic-severity, that is, participants with the least severe tics also had largest deviations, and the authors thus interpreted their findings as compensatory phenomena. Although the YGTSS has good psychometric properties [32], and one study has demonstrated consistency between two YGTSS administrations separated by time and completed by different interviewers [68], it is a mere clinical measure and none of the studies report any reliability ratings, which must be regarded a general limitation.

*4.1.2. Age*

Although the search was limited to studies that mainly included children and adolescents with TS, some included individuals with TS throughout lifetime and most show that correlations with age play an important role. Large samples that included children and adults with TS and age-matched controls deriving from a general population show prominent interactions of diagnosis with age in most regions of the brain [45,47,53] with the exception of the basal ganglia [48]. Neuroimaging studies to date support findings from clinical studies suggesting that adults with TS are a subsample of all individuals affected with TS, who have not undergone the adaptive processes that lead to an attenuation of tics in the majority of cases. However, this evidence is merely based on cross-sectional studies, in the absence of longitudinal studies.

*4.1.3. Comorbidity*

Rates of comorbidity with OCD in TS patients exceed 40% in clinical samples of adults [29,70]. Comorbidity with ADHD is observed in 60% of children with TS in clinical samples [58]. Evidence from neuroimaging studies that included both individuals with TS alone and individuals with TS and comorbid ADHD suggest that comorbid ADHD does not significantly alter the primary findings in samples of individuals with TS, thus providing support for the hypothesis of a shared genetic vulnerability as expressed in deviations of brain morphology in these comorbid conditions [52]. The extant literature suggests that comorbid OCD does not have an explicit influence on brain activation or morphology, though most studies have controlled for the presence of comorbid disorders in their statistical models. Moreover, analyses restricted to all individuals with "pure" TS and the control group, as performed in [3,37,38,45–47,51,53], have helped to confirm that primary findings were not driven by comorbid conditions.

*4.1.4. Medication*

Most of the selected studies have included children on tic-suppression medication, with the potential that these pharmacological agents may facilitate or inhibit the development of adaptive processes or influence functional and anatomical differences. Although several of the agents may have unwanted side effects along with alleviating tics, it is unlikely that the medication status of children with TS has influenced the primary findings, as most authors consider medication as a potentially confounding variable [37,38,45,47,51,53] (though see [40], where effects of medications were apparent). This is in contrast to other neuropsychiatric disorders, such as ADHD, where subgroups of patients on or off medication have showed differences in brain morphology [24,66] and in longitudinal development [64]. The general lack of evidence for changes due to medication may either be a real absence of effect, or alternatively represent small effect sizes in small samples and even smaller subgroups on the different agents. Larger studies should thus attempt to map the effects of medication on brain anatomy and function in parallel to running psychopharmacological studies testing the effectiveness of the agents used to treat tics.

*4.1.5. Intelligence*

IQ plays a crucial role for the capacity of adaptation and the general psychosocial outcome in children with neuropsychiatric disorders. Several studies indicate an IQ lower than the population mean in children with TS [12,43], whereas other studies have not confirmed this notion [34,65], potentially due to differences in sample selection and recruitment. In the studies selected for the review, children with TS did not show a lower IQ than the mean, although in some instances still a lower IQ than the control sample (see Tables 1 and 2). Hence their higher IQ may represent a positive selection for the presence of plastic processes.

*4.2. Study characteristics*

*4.2.1. Study-design*

All studies are cross-sectional in their design, which limits their ability to infer to developmental processes [30]. The core characteristic of compensatory processes is the development over time, whereas cross-sectional studies may in best case enhance the understanding of correlations with age.

*4.2.2. MRI methodology*

The techniques used to collect and analyze data play an important role for identifying deviations that may represent functional or anatomical correlates of compensatory processes. The studies presented here include anatomical and functional MRI studies, yet they use different methods of acquisition and analyses, which makes it difficult to combine the findings in a meta-analysis. Despite the wide array of methods used to explore the signs for plastic changes in children with TS, there is impressive convergence on cortico-striatal pathways, and it is possible to pinpoint processes and areas involved in neural plasticity.

## 5. Future directions

The presented studies provide evidence for the existence of ubiquitous compensatory phenomena in children with TS. All findings, however, are of correlational nature and consist most typically of deviations in brain activation or morphology that correlated with a clinical measure – tic-severity. The interpretations concerning the compensatory changes put forward by the authors lack causational relation in the absence of underlying ultrastructural components. In future studies it will be crucial to measure a child's capacity of self-regulatory control in TS, both in terms of understanding whether the capacity of self-regulatory control may be a protective factor for developing symptoms or for the ability to modulate tics.

Longitudinal imaging of representative samples of children at high risk or of younger children with TS will in the future hopefully provide clues to understanding differences in the trajectories of brain development in children with TS and provide a valid approach for disentangling causes from compensatory effects. Imaging of children who are at risk for developing TS with a combination of several MRI modalities, including anatomical MRI, functional MRI, DTI, spectroscopy, and possibly concurrent EEG recordings, will aid the pathophysiological interpretation of imaging findings in TS, supplying complementary views of brain structure and function that together will be more helpful in identifying endophenotypes for disease vulnerability that are independent of the compensatory effects of having TS with the typical symptoms of the illness [20]. A prospective longitudinal follow-up of children at high-risk that would permit scanning the sample at multiple time points would help to distinguish the trait vulnerabilities to changes related to symptom exertion and to compensatory changes that emerge early in the course of the disorder. Moreover, combining longitudinal studies with randomized clinical trials, such as trials of cognitive or behavioral exercises that are designed to encourage tic modulation, may provide the experimental control to determine whether evidence from cross-sectional studies is validated in rigorous experimental settings. In consideration of future developments, the technical advances in imaging and image processing will likely begin to bridge the gap from research to clinical applications. MRI scans may become sensitive at the level of the individual, permitting comparisons of brain activation and anatomy that may confirm or oppose a given clinical diagnosis [22].

## Acknowledgment

This work was supported in part by Research Council of Norway (grantnr. 544/H10), by a grant from the Mood Network, Health Authorities of West Norway and the Capital Region Psychiatry, Denmark. We thank Elena A. Allen for helpful discussions.
